# Quantifying mechanisms in neurodegenerative diseases (NDDs) using candidate mechanism perturbation amplitude (CMPA) algorithm

**DOI:** 10.1186/s12859-019-3101-1

**Published:** 2019-10-11

**Authors:** Reagon Karki, Alpha Tom Kodamullil, Charles Tapley Hoyt, Martin Hofmann-Apitius

**Affiliations:** 1 Department of Bioinformatics, Fraunhofer Institute for Algorithms and Scientific Computing (SCAI), Schloss Birlinghoven, 53754 Sankt Augustin, Germany; 2 Rheinische Friedrich-Wilhelms-Universität Bonn, Bonn-Aachen International Center for IT, Endenicher Allee 19a, 53115 Bonn, Germany

**Keywords:** Alzheimer’s disease, Parkinson’s disease, Mitochondrial dysfunction, Aggregation of neurofibrillary tangles, OpenBEL

## Abstract

**Background:**

Literature derived knowledge assemblies have been used as an effective way of representing biological phenomenon and understanding disease etiology in systems biology. These include canonical pathway databases such as KEGG, Reactome and WikiPathways and disease specific network inventories such as causal biological networks database, PD map and NeuroMMSig. The represented knowledge in these resources delineates qualitative information focusing mainly on the causal relationships between biological entities. Genes, the major constituents of knowledge representations, tend to express differentially in different conditions such as cell types, brain regions and disease stages. A classical approach of interpreting a knowledge assembly is to explore gene expression patterns of the individual genes. However, an approach that enables quantification of the overall impact of differentially expressed genes in the corresponding network is still lacking.

**Results:**

Using the concept of heat diffusion, we have devised an algorithm that is able to calculate the magnitude of regulation of a biological network using expression datasets. We have demonstrated that molecular mechanisms specific to Alzheimer (AD) and Parkinson Disease (PD) regulate with different intensities across spatial and temporal resolutions. Our approach depicts that the mitochondrial dysfunction in PD is severe in cortex and advanced stages of PD patients. Similarly, we have shown that the intensity of aggregation of neurofibrillary tangles (NFTs) in AD increases as the disease progresses. This finding is in concordance with previous studies that explain the burden of NFTs in stages of AD.

**Conclusions:**

This study is one of the first attempts that enable quantification of mechanisms represented as biological networks. We have been able to quantify the magnitude of regulation of a biological network and illustrate that the magnitudes are different across spatial and temporal resolution.

## Background

In recent years, systems biology approaches have played a pivotal role in the integration of multi-scale and multi-modal aspects of diseases. Knowledge assembly, one of the key outcomes of systems biology, connects entities such as genes, proteins, chemicals, miRNAs, genetic and epigenetic variants, biological processes, and phenotypes of a disease. These are represented as a set of biological networks with edges defining the types of relationships between the entities. Pathway databases such as KEGG [1], Reactome [2], and WikiPathways [3] have undertaken massive efforts of extracting and encoding biological information from the published literature to graphically depict complex biological networks as pathways. They serve as a repository of protein-protein interactions (PPIs), metabolic pathways, signal transduction pathways, cell-cell signaling pathways, and other cellular processes. They have been regarded as comprehensive knowledge assemblies for functional interpretation of genomics and provide information about characteristics, progression and aetiology of a disease. A total of 521, 2176, and 2677 pathways are represented in KEGG, Reactome, and WikiPathways respectively. These databases provide pathways in standard formats (e.g., Systems Biology Markup Language (SBML) [4] and Biological Pathway Exchange (BioPAX) [5]), enabling easy exchange of data and implementation into algorithms for visualization, simulation and analysis [6].

However, pathway databases do have some limitations. Firstly, they lack context specific representation of knowledge when it comes to disease specificity. Pathways are generalized representations of established cascade of events within a specific pathway boundary. For example, the insulin signaling pathway in KEGG draws from experimental evidence from different diseases including diabetes [7], cancer [8], and hamartoma syndrome [9]. Moreover, pathways are abstractions that have been delineated arbitrarily and do not necessarily represent pathophysiology processes (e.g., the crosstalk between insulin signaling pathway and neurotrophin signaling pathway) [10]. Secondly, the spectrum of biological information captured by pathways is limited. They are mostly populated with proteins, making them uni-modal content wise. They completely lack representation of biomarkers, genetic variations, epigenetics (genetic modifications), neuroimaging, and clinical features. For example, the Parkinson’s disease (PD) network in KEGG does not include many significant entities which play a crucial role in PD, such as the methylation of KCNH1 [11], the rs393152 variant in CRHR1 [12], and S87 SNCA phosphorylation [13]. Moreover, the fact that the map has been developed by retrieving information from 20 scientific articles (with the latest citation from 2013) infers that it is not up-to-date and incomplete [14]. Lastly, pathways are neither species, tissue, nor cell type specific. The representations in pathway databases are derived from various organisms (e.g., human, mouse, rat, and drosophila) where each species is indicated by differently colored nodes. However, interactions at the molecular level in a pathway can differ in these conditions. A study by Seok et al. (2013) reported on poor recapitulation of genomic responses of human inflammatory diseases in mouse models [15]. Warren et al. (2015) re-confirmed essential differences between these two species at the molecular level by showing that mouse models mimicked only 12% of the genes dysregulated in human conditions [16]. These studies clearly suggest that entities involved in pathways can be specific to species, tissue, cell types, and especially diseases.

Lately, there have been a few independent studies suggesting that a disease-specific mechanism differ from the canonically represented pathways in KEGG or Reactome. Kodamullil et al. (2015) have illustrated two different mechanisms on how the neurotrophin signaling pathway is regulated under normal conditions and AD [17]. Furthermore, Karki et al. (2017) have mechanistically represented the crosstalk between the insulin signaling pathway and neurotrophin signaling pathway, explaining the underlying comorbid association between AD and Type 2 Diabetes Mellitus (T2DM) [10]. Disease specific knowledge representations have improved significantly over the years due to the advancement in resources, frameworks and aforementioned limitations in the pathway databases. Several frameworks such as SMBL, GeneMania, Malacards, and OpenBEL, developed with either pathway-centric or integrated molecular network or knowledge graph approaches, are capable of representing knowledge at extent of their own features and advantages [18]. Nevertheless, these frameworks share the drawback of lacking a strategy to rank and prioritize pathways and mechanisms (i.e., knowledge sub-graphs) with the existing pathway databases. The selection of important individual graphs is often influenced by literature bias or expert’s opinion. A scoring schema that takes in to account measurable biological entities will enable researchers to overcome any biases and identify important mechanisms involved in a disease.

Several algorithms have been proposed to use pathway databases to assist in the interpretation of high-throughput *-omics* data. Drier et al. (2013) introduced the Pathifier algorithm to score dysregulated pathways in tumor samples [19]. While it is able to transform gene level information to pathway level information, it does not take into account the polarity of relationships (i.e. increase or decrease) between the genes involved. Catlett et al. (2013) devised Reverse Causal Reasoning (RCR), a reverse engineering method to detect mechanistic hypotheses from molecular profiling data that generates and scores hypothesis networks (HYPs) i.e., literature-derived causal networks consisting of an upstream node and its first downstream neighbors [20]. Similarly, Martin et al. (2014) proposed the Network Perturbation Amplitude (NPA) algorithm to assess HYPs using high-throughput measurement data and demonstrated its ability to quantify TNF-induced perturbation of inflammatory signaling [21]. Although the RCR and NPA algorithms consider both the expression levels of genes and the relationship types between genes in a network, they have the following limitations: 1) the applications are restricted to interpret treatment-induced and dose-dependent changes in activity, 2) the size of the network is too small as it only accounts for the first neighbors and 3) the interlink between HYPs (i.e. one HYP being regulator of another HYP) is not considered.

Molecular mechanisms associated with a disease are often complex; they contain cascade of events regulated by biomolecules which collectively influence biological processes and signaling pathways. Therefore, considering disease mechanisms we should be able to quantify them beyond HYPs or a network with few levels of neighbors (i.e. first and second neighbors). In fact, several cross-linked HYPs can form a basis for larger networks representing models of pathological events or disease mechanisms. Therefore, it is of the utmost importance to extend interplays between entities from HYPs to biological process, biological process to pathways, and pathways to mechanisms. Additionally, as genes tend to express differentially in different bodily regions or stages of a disease, the mechanisms in which they participate can be upregulated or downregulated by combined effect of the differentially expressed entities. To address these limitations, we have developed an extension to the NPA algorithm which is able to quantify mechanisms by scoring all of their constituent entities. As a case study, we ran the algorithm over two mechanisms (i.e. mitochondrial dysfunction in PD and aggregation of neurofibrillary tangles (NFTs) in AD) after mapping with gene expression datasets. The main objective of the study is to find out if mechanisms are regulated with different intensities as a consequence of differentially expressed genes at several resolutions.

## Results

In this study, we have deployed the CMPA algorithm on two mechanisms, one each from PD and AD. This has allowed us to quantify perturbed mechanisms and show that the amplitude of the perturbations are affected by the differentially expressed genes. Moreover, the algorithm is able to handle mechanistic information at spatial and temporal resolution.

### Mitochondrial dysfunction in PD

**Fig. 1:**
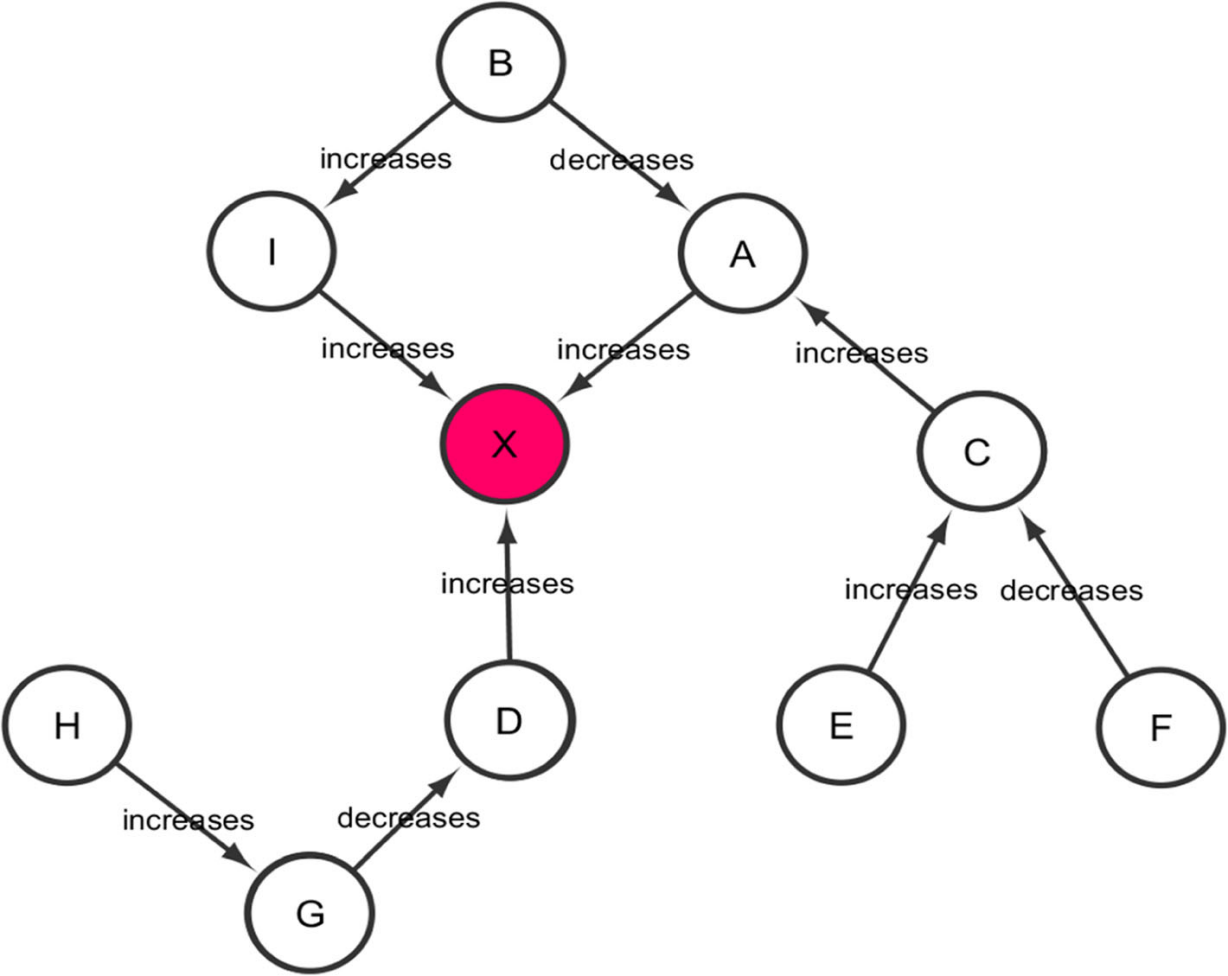
Mechanisms perturb with different intensities: **a**, **b** and **c** show the amplitude of mitochondrial dysfunction in PD across age-groups, PD stages and brain regions respectively. The CMPA scores observed to be high in age-group 40–50, Braak Stage 5–6 and cortex of PD patients. Similarly, **d** shows the perturbation of aggregation of NFTs in AD across different stages of AD. The CMPA scores are observed to be directly proportional with stages of AD

The CMPA analysis of mitochondrial dysfunction in different age-groups of PD patients depicts that the mechanism is perturbed the most in age-group 40–50 when compared to other age-groups (Fig. 1a). The magnitude of perturbation calculated as CMPA score is 4.8. Supporting this result, Lesage et al. (2016) implicate the role of mitochondrial dysfunction in the early onset of PD. Similarly, Fig. 1b shows the highest perturbation of mitochondrial dysfunction in Braak 5–6 stage of PD patients with CMPA score of 4.9. In contrast, Braak Stages 1–2 and 3–4 show less perturbation or no perturbation with CMPA scores of 0.93 and 0.08, respectively. A study by Hattingen et al. (2009) supports the role of mitochondrial dysfunction in both early and advanced stages of PD. This shows that our results (Fig. 1a and b) are in concordance with other independent studies performed at the patient level. Interestingly, it can be seen in Fig. 1a and b that the amplitude of perturbation is low in age-group 50–60, 60–70, and Braak Stage 3–4. The rationale for these observations may be due to immunity triggered recovery or/and effect of drug used for treatment of PD. The inefficacy of both the immune system and the drug might be the reason for increased mitochondrial dysfunction in Braak 5–6 Stage of PD. Furthermore, Fig. 1c illustrates that the degree of perturbation of mitochondrial function varies across brain regions of PD patients. With CMPA score of 3.3, cortex is the region of the brain with the highest mitochondrial dysfunction. The magnitude of dysfunctions in other brain regions such as the cerebellum, medulla and striatum are minimal in comparison [22]. In this context, several animal and human based studies have previously confirmed prevalence of mitochondrial dysfunction in cortex [23–25].

### Aggregation of NFTs in AD

The CMPA scores calculated for different stages of AD as shown in Fig. 1d suggests that the intensity with which aggregation of NFTs is regulated depends upon the stage of AD. The CMPA scores of incipient, moderate and severe AD are 3.6, 8.2 and 16.5 respectively. It can be clearly observed that the CMPA scores are directly correlated with the stages of AD. This comprehensively alienates with the findings of increased NFT burden with the progression of AD as reported by several studies [26–28].

## Discussion

As the NeuroMMSig server embeds numerous molecular signatures implicated in AD and PD, it provides us the opportunity to extend the CMPA analysis beyond the two mechanisms we have undertaken in this study. An extensive implementation of the CMPA algorithm on NeuroMMsig based mechanisms will enable us to rank mechanisms based on the CMPA scores. By scoring mechanisms on several resolutions, we may be able to prioritize the targetable mechanisms and thereby decide on the best suited medicine. For example, the CMPA score of 0.08 for mitochondrial dysfunction in a PD patient of Braak Stage 3–4 suggests reduced perturbation of the mechanism. Hence, targeting dysfunctional mitochondrial activity for patients with Braak 3–4 stage of PD might not be as important as it is for Braak 5–6 stage of PD. This sort of approach defies any literature bias, where one mechanism can be overly represented in a knowledge network because of the high density of supporting publications.

### CMPA scores are mechanism specific

It has been observed that the CMPA scores are unique for all the gene expression datasets used in this study. Therefore, for each sub-groups of these datasets we have essentially been able to show that mechanisms are regulated with different magnitudes. The one sample t-test for GSE57475’s age-group 40–50 in PD rejected the null hypothesis with a *p*-value < 2.2e-16 and *t*-statistic of − 166. The mean of 10,000 CMPA scores was 0.19 as compared to the actual CMPA score of 4.8. Similarly, the null hypothesis for GSE28146’s moderate sub-group of AD was also rejected as the mean of CMPA scores and actual CMPA score were 1.77 and 8.2 respectively. Therefore, the alternative hypothesis i.e., true mean is not equal to 8.2 was favored with a *p*-value < 2.2e-16 and *t*-statistic of − 67.19. These results suggest that the CMPA score obtained from the real gene expression values is unique to a mechanism and is highly unlikely to occur just by chance.

## Conclusions

In this study, we have demonstrated that blending computable knowledge and data in a given disease context provides us with new options for inference. Although strategies to integrate knowledge driven and data driven approaches already exist, our work deals with two new aspects: Firstly, we have been able to quantify candidate mechanisms underlying diseases. This is novel when compared to previous studies because we claim that our work is one of the first attempts to score complex biological networks that explain disease etiology. The causal relationship in OpenBEL, which forms the basis of making the OpenBEL knowledgebase computable, is the key in devising the CMPA algorithm. Without the information on the causality of the interacting biological entities, measuring the amplitude of a regulated mechanism is not possible. Secondly, we could demonstrate that differentially expressed genes regulate their corresponding mechanisms with different intensities. The differences in regulation intensities of mechanisms in temporal and spatial resolution have been reported through our study for the very first time. Based on the CMPA algorithm applied on 3 selected GE datasets, we observed that PD patients of Braak Stage 5–6, the age-group 40–50 and the cortex region of the brain have high magnitudes of mechanism perturbation. Similarly, we found out that the magnitudes of perturbation of aggregation of NFTs in AD increase with the progression of AD. From our results, we can conclude that the classical approach of associating mechanisms to progressive disorders can be improved by quantifying and prioritizing specifics such as disease stages, patient groups and brain regions.

## Methods

### Construction of mechanistic NDD knowledgebase

The unstructured textual information containing cause-and-effect or correlative relationships from literature specific to AD and PD were encoded as triples (i.e. subject-predicate-object) using OpenBEL. Furthermore, the triples are enriched with meta-annotations such as cell type, species, anatomy and stage of the disease. With additional curation efforts, each triple was assigned to a particular mechanistic sub-graph as described by Domingo-Fernandez et al. (2017) [29]. The resulting sub-graph contains several inter-connected triples depicting a disease mechanism. A total of 124 and 65 molecular mechanisms specific to AD and PD respectively are integrated in NeuroMMSig. For our analysis, we have taken into consideration the mechanisms depicting aggregation of neurofibrillary tangles (NFTs) in AD and mitochondrial dysfunction in PD. The mitochondrial dysfunction in PD is considered as one of the most important mechanisms associated with the PD etiology. Moreover, the AETIONOMY project (www.imi.europa.eu/projects-results/project-factsheets/aetionomy) has selected this mechanism for its intensive research. Similarly, the aggregation of NFTs in AD is a well-known AD phenotype and regarded as an important hypothesis in AD etiology. After filtering the mechanisms for causal relationships manually and using a threshold of five nearest neighbors as network size, the mechanism representing aggregation of NFTs in AD had a total of 31 nodes and 57 edges while the mitochondrial dysfunction in PD had 35 nodes and 54 edges (Additional file 1).

### Selection of datasets as a scoring input

This study aims to quantify the intensity of perturbed mechanisms associated with diseases as the consequence of differentially expressed genes. Therefore, the candidate mechanism perturbation amplitude (CMPA) algorithm reduces the existing caveat of mere mechanism-disease associations by showing that mechanisms regulate with different intensities across spatial and temporal dimensions. Gene expression datasets from GEO (Gene Expression Omnibus) were selected such that the expression profiles could be categorized based on spatial dimensions (i.e., brain regions), temporal dynamics (i.e., age groups) or stages of the disease. These datasets were analyzed using GEO2R from GEO. A brief description of each of the datasets is given below:

I. **GSE49036** - Samples from Substantia nigra of different Braak Stages PD patients
II. **GSE57475** - Blood transcripts of PD patients of 4 different age groups
III. **GSE28894** - Samples from cerebellum, medulla, cortex, and striatum of PD patients
IV. **GSE28146** - Samples from Hippocampus of different stages of AD patients

### Implementation of candidate mechanism perturbation amplitude (CMPA) algorithm

The strategy involved in this study is to integrate knowledge driven approaches and data driven approaches to score biological networks. Here, we have used gene expression profiles mapped to NeuroMMSig based causal networks to calculate the extent of perturbation of mechanism associated with AD and PD. A total of 3 datasets (i.e., GSE49036, GSE57475 and GSE28894) were mapped to the causal network representing mitochondrial dysfunction in PD while GSE28146 was mapped to the network representing aggregation of NFTs in AD. The causality between biological entities captured in BEL is one of the special features of BEL which many of the pathway representations are void of. Without the information about causal edges in disease networks, devising a scoring algorithm is not possible.

#### Scoring function

The expression profiles (i.e., log fold change values) are assigned as weights to the genes involved in a mechanism. The directionality of edges is taken from the mechanistic causal network as + 1 for increase and − 1 for decrease. A scoring function implemented in Python uses the weights and directionality of edges to quantify the amplitude of dysregulated mechanisms. A positive score implies that a particular mechanism for a given dataset is upregulated (i.e., perturbed) due to the interplay of involved downstream entities. Likewise, a negative score indicates that the mechanism is downregulated while a score of zero suggests no change in the mechanism.

#### Perturbation amplitude

**Fig. 2:**
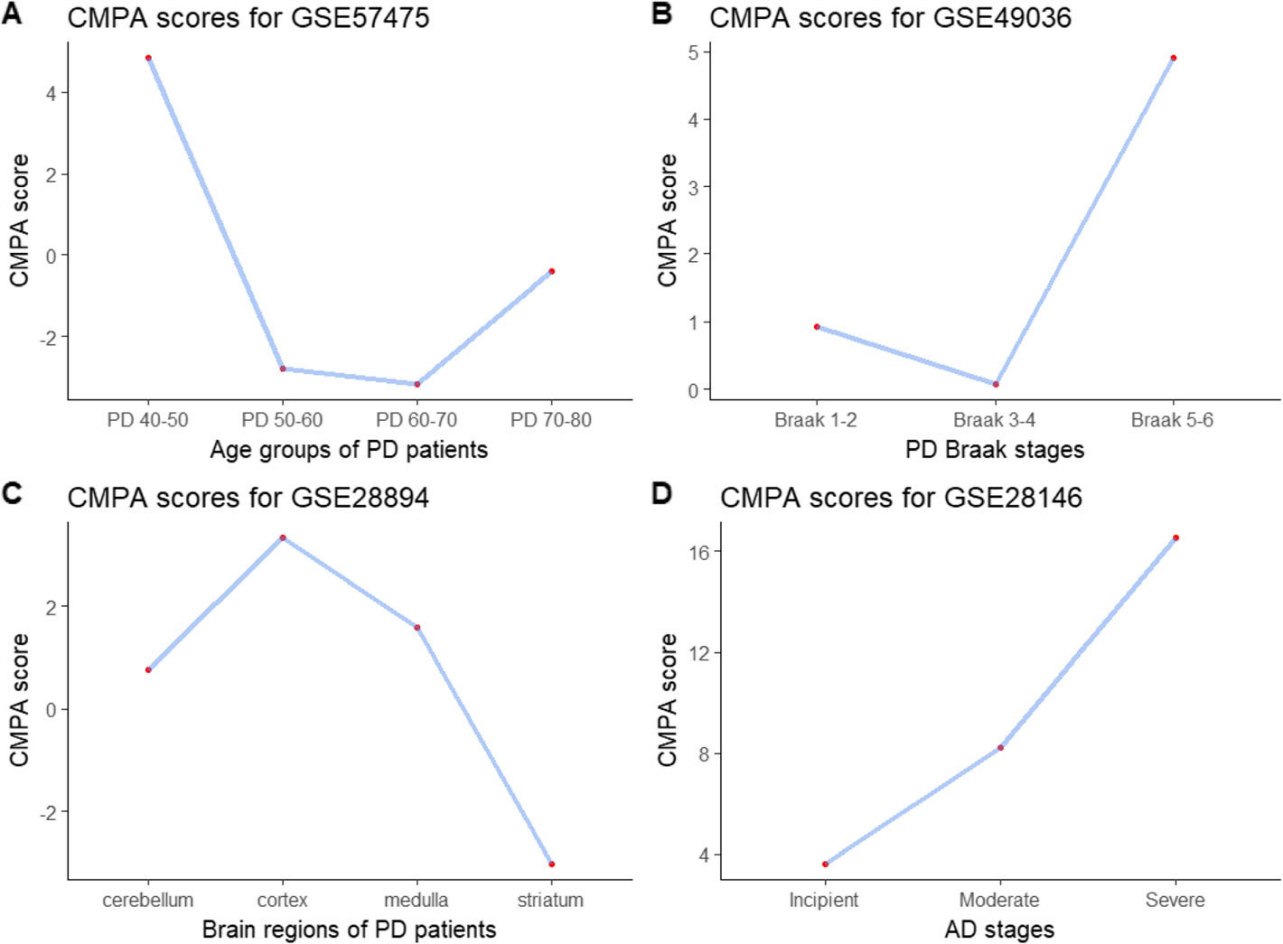
A general biological network: A schematic representation of a mechanism where several upstream nodes (either genes/proteins or biological processes) converge to a centrally located node X (highlighted in red)

The amplitude of perturbation is calculated for the central node (most upstream node) in the network to which several downstream nodes are connected. These downstream nodes can be either direct or indirect neighbors of the central node. Moreover, a downstream node can be a child node for other upstream nodes. Figure 2 illustrates a general cause-and-effect mechanism where downstream nodes converge to the centrally located node (node X, highlighted in red). The final score of the central node is calculated by enumerating the effect of differentially expressed downstream nodes on a particular mechanism context (in this case, the central node and the scored downstream nodes).

After this, the nodes outgoing from the central node were not considered (filtered and removed) as the central node mostly connects only either to another hub of the knowledgebase (in our case: Parkinson Disease) or to another central node (which can be another mechanism) and need not be scored.

The following *pseudocode* implemented in python was used to calculate the perturbation (CMPA) scores.

- Identify and create a list of hubs (H) in the network i.e. a node with several incoming and outgoing edges
- For each hub in H
  - If hub **has incoming** edges from another hub from the list H
    - Skip
  - If hub has **no incoming** edges from another hub from list H
    - Calculate Impact Factor (IF)
      IF = hubWeight + $$ \sum \limits_{i=1}^N{S}_i.{\beta}_i $$
      where,
      *S*_*i*_ = Sign of the edge (+ 1 for increase, − 1 for decrease)
      *ß*_*i*_ = Log_2_ fold change value
      *N* = number of incoming nodes
    - Remove hub from H
- Calculate CMPA score
  - CMPA score = $$ \sum \limits_{j=1}^M{IF}_i $$
    Where,
    *M* = number of hubs

The CMPA algorithm is devised such that it is able to quantify the overall effect of differentially expressed entities involved in a cause-and-effect model of a disease mechanism. The algorithm functions on a simple logic that downstream nodes pass their values to the connected upstream nodes. For example, the value of H is passed to X through H – G – D – X (Fig. 1). In doing so, it is assured that G gets a value from H before G passes its value to D. The nodes G and D are hub nodes in the network because they have incoming and outgoing edges. For each hub node in the network, a score called Impact Factor (IF) is calculated. The sum of all the IFs, represented as CMPA score, quantify the amplitude of perturbation of a mechanism.

##### Statistical assessment of CMPA scores

The CMPA scores generated by the CMPA algorithm are expected to be unique for each gene expression dataset. This is because of the distinct property of each gene responding differently to different conditions. However, a CMPA score can be considered absurd if it remains unchanged after random sampling of genes and their expressions. In the case differences in CMPA scores are observed between CMPA analyses performed with actual gene expressions and randomized gene expressions, it can be concluded that the CMPA score is specific to a mechanism and represents the true magnitude of its perturbation. This was assessed by first performing a permutation (number of permutations = 10,000) where each gene was assigned a random gene expression value from the pool of real gene expression values. Afterwards, the CMPA algorithm was implemented to each of the permuted samples. Lastly, one sample Student’s t-test was conducted with the null hypothesis that the mean of 10,000 CMPA scores is equal to the actual CMPA score. If the resulting *p*-value is below the threshold of 0.05, then the null hypothesis is rejected in favor of the alternative hypothesis.

## Supplementary information

**Additional file 1: Figure S1.** Mitochondrial dysfunction in PD manifests as a consequence of increased oxidative stress and endoplasmic reticulum stress and decreased regulation of mitophagy. **Figure S2.** The aggregation of NFTs in AD is triggered by the insulin receptor signaling pathway and several genes that destabilize MAPT activity.

## Data Availability

1. GSE57475 - https://www.ncbi.nlm.nih.gov/geo/query/acc.cgi?acc=GSE57475 2. GSE49036 - https://www.ncbi.nlm.nih.gov/geo/query/acc.cgi?acc=GSE49036 3. GSE28894 - https://www.ncbi.nlm.nih.gov/geo/query/acc.cgi?acc=GSE28894 4. GSE28146 - https://www.ncbi.nlm.nih.gov/geo/query/acc.cgi?acc=GSE28146

## Abbreviations

AD: Alzheimer’s Disease
BioPAX: Biological Pathway Exchange
CMPA: Candidate Mechanism Perturbation Amplitude
GEO: Gene Expression Omnibus
NFT: Neurofibrillary Tangle
NPA: Network Perturbation Amplitude
PD: Parkinson’s Disease
RCR: Reverse Causal Reasoning
SMBL: Systems Biology Markup Language
