# Supplementary material for: Quantifying mechanisms in neurodegenerative diseases (NDDs) using candidate mechanism perturbation amplitude (CMPA) algorithm
Source: BMC Bioinformatics. 2019 Oct 11;20:494. doi: 10.1186/s12859-019-3101-1 (PMC6788110; doi:10.1186/s12859-019-3101-1)
Supplement: Supplementary file 1 — Additional file 1: Figure S1. Mitochondrial dysfunction in PD manifests as a consequence of increased oxidative stress and endoplasmic reticulum stress and decreased regulation of mitophagy. Figure S2. The aggregation of NFTs in AD is triggered by the insulin receptor signaling pathway and several genes that destabilize MAPT activity. [file 12859_2019_3101_MOESM1_ESM.docx]

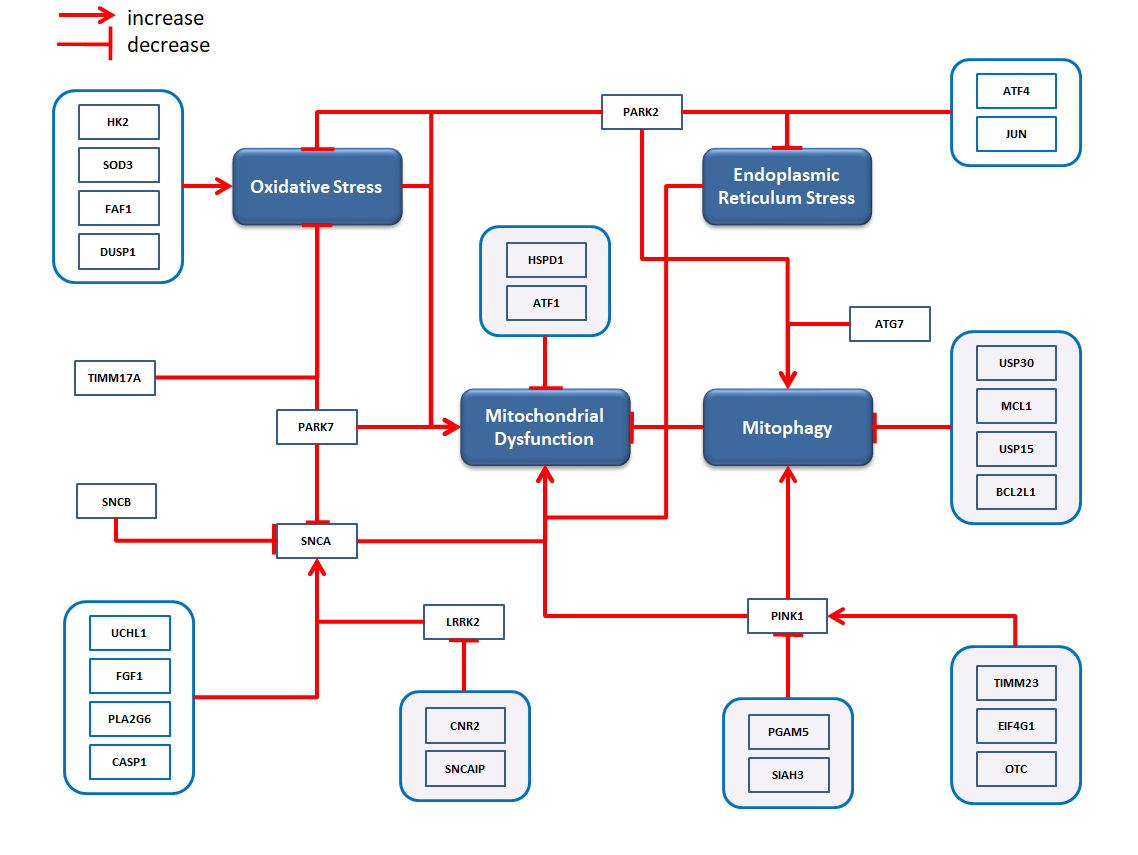


**Figure S1**. *Mitochondrial dysfunction in PD* *manifests as a consequence of increased oxidative stress and endoplasmic reticulum stress and decreased regulation of mitophagy.*


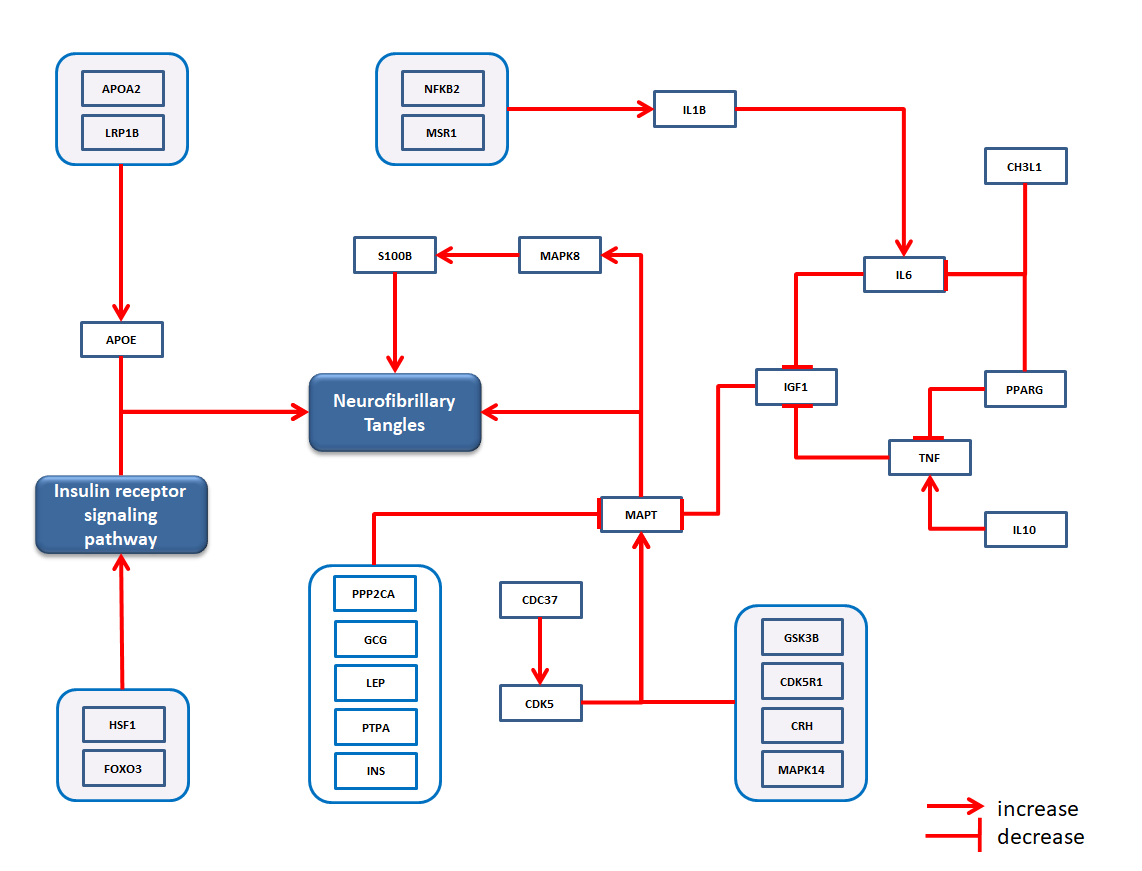


**Figure S2**. *The aggregation of NFTs in AD is triggered by the insulin receptor signaling pathway and several genes that destabilize MAPT activity.*
